# Neuroleptospirosis in patients presenting with primary central nervous system infection: A multicentre study

**DOI:** 10.1371/journal.pntd.0014183

**Published:** 2026-04-07

**Authors:** Nilanka Perera, Champika Gunawardena, Varuna Abeyratne, Parami Rajapakse, Thisal Semina, Wimalasiri Uluwattage, Asanka Ratnayake, Suresh Mendis, Nandana Dickmadugoda, M. Mujaheith, Lilani Karunanayake, Udaya K. Ranawaka

**Affiliations:** 1 Department of Medicine, Faculty of Medical Sciences, University of Sri Jayewardenepura, Nugegoda, Sri Lanka; 2 Neurology Unit, Teaching Hospital, Ratnapura, Sri Lanka; 3 Professorial Medical Unit, Colombo South Teaching Hospital, Kalubowila, Sri Lanka; 4 National Hospital Galle, Karapitiya, Sri Lanka; 5 National Reference Laboratory for Leptospirosis, Medical Research Institute, Colombo, Sri Lanka; 6 Department of Medicine, Faculty of Medicine, University of Kelaniya, Ragama, Sri Lanka; Yale University School of Medicine, UNITED STATES OF AMERICA

## Abstract

**Background:**

Leptospirosis is well-known to cause neurological involvement. However, data on the frequency of neuroleptospirosis as a cause of primary central nervous system (CNS) infections is sparse.

**Methodology/principal findings:**

An observational study was conducted in three regions in Sri Lanka between October 2021-March 2024. Patients with a primary diagnosis of meningitis, meningoencephalitis or encephalitis were recruited. Blood and cerebrospinal fluid (CSF) were analysed for pathogenic *Leptospira* DNA by real-time PCR, and serology by microscopic agglutination test (MAT) on admission and after 10–14 days. A confirmed case was a patient with evidence of CNS infection with positive CSF or blood PCR and/or 4-fold rise/single titre ≥1:320 in MAT. A total of 100 patients were studied with a median age of 55 (IQR 39.7 – 69.7) years and 52 (52%) males. Suspected clinical diagnoses were meningitis (n = 66,66%), meningoencephalitis (n = 22,22%) and encephalitis (n = 12,12%). Eleven (11%) patients were laboratory-confirmed as neuroleptospirosis; meningitis (n = 3), meningoencephalitis (n = 3), encephalitis (n = 5). Three patients had positive blood *Leptospira* PCR, one had positive blood and CSF *Leptospira* PCR, three had high initial MAT and four had a 4-fold rise in MAT. Neurological manifestations included headache (n = 8), seizures (n = 2), confusion (n = 7), photophobia (n = 4), neck stiffness (n = 6) and limb weakness (n = 5). Majority of patients with positive blood or CSF PCR for *Leptospira* (n = 3, 75%) did not have a CSF cellular reaction. The neuroleptospirosis group had a lower requirement for intensive care compared to other CNS infections (9% vs 13.5%) and recovered without residual neurological disability. There were no deaths in the neuroleptospirosis group compared to 11 (12.4%) deaths in other CNS infections.

**Conclusions:**

Leptospirosis is an important cause of primary CNS infections, and may lead to more favourable outcomes. It is important to consider leptospirosis as an aetiological agent in the laboratory workup of primary CNS infections in leptospirosis-prevalent settings.

## Author summary

Leptospirosis is a leading zoonotic disease in the world with high morbidity and mortality. Diverse neurological manifestations occur following this spirochaetal infection. Central nervous system (CNS) manifestations range from meningitis, encephalitis, autoimmune encephalitis, acute disseminated encephalomyelitis, cerebritis to cranial arteritis. However, neuroleptospirosis is not considered a common aetiological cause of primary CNS infections in many countries. Sri Lanka is endemic for leptospirosis with a high disease burden. We conducted a multicentre study to identify the occurrence of neuroleptospirosis in patients presenting with a primary CNS infection. There were 11 neuroleptospirosis patients among 100 patients who presented with encephalitis, meningitis or meningoencephalitis. Primary neuroleptospirosis patients had similar clinical presentations and baseline laboratory investigations compared to other CNS infections. Neuroleptospirosis was presumed to be due to direct bacterial invasion in the acute phase in addition to immune-mediated aseptic meningitis in the immunological phase. In our cohort, neuroleptospirosis patients had a more favourable outcome. This study highlights the importance of considering primary neuroleptospirosis as a possible aetiology in patients presenting with features of a CNS infection and performing appropriate microbiological investigations in endemic settings.

## Introduction

Leptospirosis is caused by pathogenic spirochetes of the genus *Leptospira* transmitted via urine of infected animals [1], and it is a leading zoonotic disease of high morbidity and mortality worldwide [2]. The burden of disease is higher in the tropics than in temperate regions, largely affecting resource-poor countries [2,3]. Leptospirosis has spread to many other regions, causing epidemics due to globalization, climate change and travel [4], highlighting the global importance of this disease.

The disease spectrum ranges from mild anicteric leptospirosis, characterized by fever and myalgia, to a more serious Weil’s syndrome manifesting as deep jaundice, acute kidney injury (AKI), and coagulation abnormalities [5–7]. Mortality in leptospirosis is mainly attributed to pulmonary haemorrhage and renal injury [2,8,9]. Neurological manifestations are seen in 10–15% of patients with leptospirosis (neuroleptospirosis) [7], and these are mainly reported in patients with other organ involvement characteristic of leptospirosis. A study conducted in South India described 31 cases of neuroleptospirosis treated over five years; all patients had hepato-renal involvement and other manifestations of leptospirosis [10]. Leptospirosis presenting with neurological involvement without typical haematological, renal or hepatic involvement (primary neuroleptospirosis) is rare. We previously reported three patients with primary neuroleptospirosis presenting without typical haematological, renal or hepatic involvement in Sri Lanka [11,12]. Several other case reports have documented the different clinical manifestations of primary neuroleptospirosis [13–19], but large case series are lacking. Leptospirosis is rarely considered in the differential diagnosis of patients presenting with features suggestive of a central nervous system (CNS) infection. The few available case reports in literature highlight the importance of a high degree of suspicion in patients presenting with features of CNS infection in endemic settings [20–24]. Identification of leptospirosis as the underlying aetiological agent of primary CNS infections would help to understand the disease pattern and clinical outcomes, and guide appropriate treatment. However, data on neuroleptospirosis in patients presenting with a CNS infection is sparse.

Published literature is limited to a few observational studies in adults [25–29]. Some previous studies have used only antibody-based methods [7,10], potentially missing early infection during the leptospiraemic phase. The frequency of neuroleptospirosis presenting as a primary CNS infection is unknown in many countries endemic for leptospirosis. We hypothesized that leptospirosis is an aetiological agent causing primary CNS infections in endemic Sri Lanka. We conducted a multicentre study to identify leptospirosis as a cause of primary CNS infection using both PCR and MAT and to describe the clinical and laboratory characteristics and outcome.

## Methods

### Ethics statement

All study participants were recruited following written informed consent. Written informed proxy consent was obtained when study participants lacked capacity to provide consent. Ethical approval was obtained from the Research Ethics Committee of University of Sri Jayewardenepura (Ref no 07/21).

### Study design

A multi-centre prospective, descriptive study was conducted from October 2021 to March 2024 in three regions of Sri Lanka with high prevalence of leptospirosis. The study was conducted in the medical and/or neurology units of three tertiary care hospitals. The Colombo South Teaching Hospital (CSTH) is situated in the Colombo district (Western province), Teaching Hospital, Ratnapura (THR) in the Ratnapura district of Sabaragamuwa province and the Colombo North Teaching Hospital (CNTH) in the Gampaha district of Western Province.

### Study procedure

Patients clinically diagnosed as having a primary CNS infection (meningitis and/or encephalitis) and investigated with lumbar puncture were recruited following informed written consent. Study participants who lacked capacity to provide consent were recruited following written proxy consent.

A diagnosis of meningitis was considered in the presence of ≥2 of following features; headache, fever >38.5^0^C, photophobia and/or phonophobia, neck stiffness with cerebrospinal fluid (CSF) leucocytes >5/μL [30]. In addition, patients with a clinical syndrome of meningitis with a positive blood/CSF culture or positive CSF/blood PCR for an organism without CSF pleocytosis were classified as “probable meningitis”, adopted from previous guidelines published by the World Health Organization [31]. A diagnosis of encephalitis was considered according to Consensus Statement of the International Encephalitis Consortium [32]. Patients who did not undergo CSF analysis, pregnant patients, children (<18 years), and patients with an alternative non-infective cause for neurological manifestations were excluded.

Blood and CSF were collected for detection of *Leptospira* DNA by real-time polymerase chain reaction (rt-PCR), and microscopic agglutination test (MAT) for *Leptospira* was performed in blood during acute infection at the time of recruitment (on admission) and 10–14 days later to detect seroconversion or a rising titre. All microbiological investigations were performed at the National Reference Laboratory for leptospirosis, Medical Research Institute, Colombo. Data on the clinical presentation and physical signs were derived from the patient by direct questioning and assessment. Laboratory investigations and outcome were extracted from patient records by trained research assistants using a pretested data extraction sheet. Patients were reassessed at 14-days after discharge to assess neurological disability or death. Data was entered into a Microsoft EXCEL sheet.

### Diagnosis of neuroleptospirosis

A diagnosis of neuroleptospirosis was established in the presence of a clinical syndrome compatible with meningitis, meningoencephalitis or encephalitis and evidence of acute leptospirosis [33] (Table 1).

**Table 1:** Diagnostic criteria used for acute leptospirosis.

Criteria
Positive for *Leptospira* DNA in blood
Positive for *Leptospira* DNA in CSF
A single MAT titer ≥1:320
A 4-fold rise in MAT titer or seroconversion in the acute and convalescent serum samples

*A diagnosis of leptospirosis was established in the presence of one or more of the above criteria.

### Statistical analysis

The data were analysed using the IBM Statistical Package for the Social Sciences (SPSS) version 27. The proportion of neuroleptospirosis patients was given as a percentage from the total study population. Descriptive statistics were used to describe demographic parameters, clinical manifestations and laboratory parameters in the study population. Categorical variables among patients with and without neuroleptospirosis were compared with the chi-square test and continuous variables were compared using the Mann-Whitney U test (skewed distribution). A significance level of p < 0.05 was used.

## Results

There were 100 patients with primary CNS infection; males 52%, median age 55 years (IQR 39–69). The neurological diagnoses were meningitis in 66 patients (66%), meningoencephalitis in 22 (22%) and encephalitis in 12 (12%). Eleven (11%) patients were laboratory-confirmed as neuroleptospirosis. Diagnosis was confirmed by either detection of *Leptospira* DNA by PCR in blood or CSF in 4 (36.3%), high MAT titer in serum in 3 (27.2%) and detection of a four-fold rise in MAT titer in paired sera in 4 (36.3%) study participants. The clinical characteristics of the study participants are given in Table 2.

**Table 2:** Clinical characteristics of the study population at presentation.

Characteristic	Neuroleptospirosis n = 11	Non-neuroleptospirosis n = 89	Significance
Age in years, median (IQR)	57 (48-67)	55 (39-70.5)	0.83
Males, n (%)	8 (72.7)	44 (49.4)	0.14
Neurological diagnosis, n (%)			
Meningitis*	3 (27.3)	63 (70.8)	**<0.01**
Meningoencephalitis	3 (27.3)	19 (21.3)	0.65
Encephalitis	5 (45.5)	7 (7.9)	**<0.001**
Symptoms, n (%)			
Fever	10 (90.9)	79 (88.8)	0.83
Myalgia	3 (27.3)	36 (40.4)	0.40
Headache	8 (72.7)	68 (76.4)	0.79
Photophobia	4 (36.4)	22 (24.7)	0.40
Phonophobia	3 (27.3)	10 (11.2)	0.14
Abdominal pain	2 (18.2)	17 (19.1)	0.94
Reduced urine output	3 (27.3)	12 (13.5)	0.23
Shortness of breath	2 (18.2)	21 (23.6)	0.69
Giddiness	3 (23.7)	22 (24.7)	0.85
Seizures	2 (18.2)	22 (24.7)	0.63
Examination findings, n (%)			
GCS, median (IQR)	13 (12-15)	14 (11-15)	0.53
Neck stiffness	6 (54.5)	49 (55.1)	0.97
Confusion	7 (63.6)	59 (66.3)	0.86
Aphasia	6 (54.5)	15 (16.9)	**<0.01**
Limb weakness	5 (45.5)	12 (13.5)	**<0.01**
Muscle tenderness	2 (18.2)	16 (18.0)	0.99
Hypotension	1 (9.1)	6 (6.7)	0.77
Tachycardia	1 (9.1)	12 (13.5)	0.68
Laboratory findings, median (IQR)			
White blood cells (x10^3^/μL)	12.4 (8.2-21)	12.0 (9-15)	0.46
Haemoglobin (g/dL)	11.3 (10.6-14.4)	12 (10.7-13.2)	0.78
Platelets (x10^3^/μL)	205 (149-371)	234 (187-301)	0.51
AST (IU/L)	33 (24.9- 61.5)	38 (25.1- 71.4)	0.87
ALT (IU/L)	21.6 (15.0-44.9)	31.8 (17.8-51.0)	0.38
Serum creatinine (μmol/L)	92.0 (81-124.5)	88.3 (79.1-118.8)	0.75
CSF analysis, median (IQR)			
Protein (mg/dL)	72.6 (41.8-89.8)	81.9 (43.6-144.2)	0.51
PMN (cells/mm^3^)	2 (0-25)	10 (3-33.5)	0.09
Lymphocytes (cells/mm^3^)	6 (0-60)	14 (5-86)	0.10
Red cells (cells/mm^3^)	36 (2-125)	34.5 (8-285)	0.51
CSF: blood glucose	0.54 (0.31-0.59)	0.56 (0.40-0.64)	0.81
Intravenous antibiotics, n (%)			
3^rd^ generation cephalosporin	10 (90.9)	81 (91.0)	0.99
Vancomycin	4 (36.4)	15 (16.9)	0.29
Meropenem	2 (18.2)	21 (23.6)	0.69
Ampicillin	1 (9.1)	10 (11.2)	0.83
Intravenous acyclovir, n (%)	7 (63.6)	51 (57.3)	0.88
Intravenous steroids, n (%)	5 (45.5)	42 (47.2)	0.91
Escalation to ICU care, n (%)	1 (9.1)	12(13.5)	0.68
Outcome, n (%)			
Complete recovery without neurological disability	11 (100)	69 (88.5)	0.23
Death	0	11 (12.4)	0.60

NS; not significant, SGOT; Serum Glutamic-Oxaloacetic Transaminase, SGPT; Serum Glutamate Pyruvate Transaminase, CSF; Cerebrospinal fluid, PMN; Polymorphonuclear leucocytes, ICU; Intensive care unit. *Confirmed meningitis and probable meningitis were classified together in the analysis.

Clinical symptoms on presentation or baseline laboratory parameters at presentation were not significantly different among patients diagnosed as neuroleptospirosis compared to non-neuroleptospirosis group except for the presence of aphasia and limb weakness. Limb weakness was seen more frequently among the neuroleptospirosis group compared to non-neuroleptospirosis group (OR 5.3, 95% CI 1.4-20.3, p < 0.05). Similarly, aphasia was seen more frequently in the neuroleptospirosis group (OR 5.9, 95% CI 1.6-21.9, p < 0.01). Of the neurological syndromes, meningitis was more common in the non-neuroleptospirosis group compared to the neuroleptospirosis group (Table 2). Meningoencephalitis and encephalitis were seen more frequently among neuroleptospirosis than non-neuroleptospirosis group (Table 2). Aphasia was more frequent among patients who had meningoencephalitis and encephalitis (n = 13, 38.2%) compared to patients with meningitis (n = 8, 12.1%) (p < 0.01). Similarly, limb weakness was seen more frequently among patients with a meningoencephalitis or encephalitis (n = 11, 32.4%) compared to meningitis (n = 6, 9.1%) (p < 0.01) among the study participants. The proportion of patients with thrombocytopaenia (platelet count <150 x 10^3^/μL) was similar among the neuroleptospirosis group compared to other CNS infections (3/11 vs 15/89, p = 0.40). We observed thrombocytopaenia in 3 neuroleptospirosis patients on admission, and one patient had thrombocytopaenia during the course of illness. Transaminitis (elevated liver enzymes above the upper limit of normal) was an initial manifestation in two patients, and in two more patients during hospital stay. Similarly, acute kidney injury (AKI) was seen in two patients on admission and in two more patients during the course of illness. Brain imaging by non-contrast computed tomography (CT) was performed in 7 patients with neuroleptospirosis and one patient had white matter oedema in left frontal lobe. Other patients had normal imaging.

The study population received different antibiotic regimens according to the local hospital protocols (Table 2). Empirical antibiotics included 3^rd^ generation cephalosporin, vancomycin for coverage of penicillin-resistant *Streptococcus pneumoniae* and ampicillin in the presence of risk factors for *Listeria monocytogenes* or meropenem. In addition to antibiotics prescribed for a primary CNS infection, 2 (18.2%) of neuroleptospirosis group received penicillin and doxycycline each. All patients received antibiotics from the day of admission. Intravenous steroids were administered to 5 (45.5%) patients. Treatment did not differ from the non-neuroleptospirosis group. Outcome (death or disability) from the infection did not differ between patients who received steroids (n = 39, 48.8%) compared to who did not (n = 8, 40%, p = 0.48). Some patients received antibiotics for secondary infections such as cannula-site infections or aspiration pneumonia as appropriate.

The need for escalation to intensive care was low in the neuroleptospirosis group and all patients made a complete recovery without residual neurological disability (Table 2). Patient 9 who was escalated to intensive care in the neuroleptospirosis group had low GCS (12/15) with multiorgan involvement (Table 3). There were no deaths in this group compared to 11 (12.4%) deaths in the ‘non-neuroleptospirosis” group (Table 2). There were none with neurological disability in the neuroleptospirosis group compared to 9 (10.1%) in the “non-neuroleptospirosis” group and all had persistent disability at 14-days of follow-up.

**Table 3:** Characteristics of the confirmed-neuroleptospirosis group (n = 11).

Case no.	Age, gender	Symptom onset (from the day of fever)	Clinical presentation	Neurological syndrome	Confirmation of Leptospirosis	CSF					Other findings
						Appearance	Protein (mg/dL)	CSF: blood glucose	PMN	Lymphocytes	
1	21, M	4	Fever, headache, seizures, limb weakness, photophobia GCS 15/15	Encephalitis	Serum PCR positive	Clear	31.2	0.75	2	11	CT brain: normal
2	48, F	4	Fever, headache, confusion, neck stiffness	Meningitis	4x MAT rise	Clear	157.4	0.25	470	260	CT brain: no cerebral oedema, bilateral otitis media
3	62, M	3	Fever, headache, confusion, seizures, photophobia and phonophobia, limb weakness, GCS 13/15	Meningoencephalitis	MAT > 1:320 (Titer 1:10240)	Clear	89.8	0.57	12	38	CT brain: normal Mild AKI
4	51, M	5	Headache, confusion, neck stiffness, aphasia, GCS 12/15	Meningoencephalitis	4x MAT rise	Clear	75.5	0.15	25	60	CT brain: normal
5	49, F	5	Fever, headache, confusion, limb weakness, aphasia, GCS 15/15 ---- 11/15	Meningoencephalitis	4x MAT rise	Clear	69.2	0.57	26	101	CT brain: white matter oedema in L/frontal lobe
6	34, M	4	Fever, headache, photophobia, phonophobia	Probable meningitis	Serum PCR positive	Clear	41.8	0.9	0	0	CRP 242 mg/L
7	64, M	15	Fever, confusion, neck stiffness, aphasia, GCS 8/15	Encephalitis	Serum PCR positive	Clear	50.1	0.59	0	0	low platelets, hepatitis, AKI after admission CRP 157 mg/L
8	73, M	3	Fever, headache, confusion, neck stiffness	Probable meningitis	Serum and CSF PCR positive	Clear	139.7	0.55	1	0	None
9	85, M	2	Fever, confusion, photophobia, neck stiffness, GCS 12/15	Encephalitis	4x MAT rise	Clear	80.1	0.31	1	0	CT brain: normal High bilirubin, low platelets, hepatitis after admission CRP 138 mg/L Managed in the ICU
10	67, M	3	Fever, limb weakness, aphasia, GCS 11/15	Encephalitis	MAT > 1:320 (Titer 1:20480)	Clear	72.6	0.58	4	6	Low platelets on admission, CRP 166 mg/L
11	57, F	7	Fever, neck stiffness, limb weakness, GCS 12/15	Encephalitis	MAT > 1:320 (Titer 1:640)	Clear	24.1	0.45	0	0	CT brain: normal AKI, cholestatic hepatitis

*CSF; Cerebrospinal fluid, PMN; Polymorphonuclear leucocytes, PCR; polymerase chain reaction, MAT; microscopic agglutination titer, CT; computed tomography,* *CRP; C-Reactive Protein, GCS; Glasgow Coma Scale, AKI; Acute kidney injury, ICU; Intensive Care Unit.*

A detailed description of the confirmed neuroleptospirosis group is given in Table 3. A majority (n = 8, 72.7%) had neurological symptoms in the first five days of illness. Although these patients in the neuroleptospirosis group had a clinical syndrome of a primary CNS infection with fever, confusion, meningeal irritation and focal neurological deficits (Table 3), some patients (n = 2, 18.2%) did not fulfil laboratory criteria for meningitis due to absence of pleocytosis in the CSF or exhibit features suggestive of encephalitis. These patients were classified as “probable meningitis”. Both these patients had positive PCR in serum, CSF or both (Table 3). This is an interesting finding and it is possible that initial *Leptospira* invasion in to CSF could have been cleared without a significant inflammation. Finding of a group with minimal CSF pleocytosis would warrant further investigation into the varied pathogenesis of neuroleptospirosis.

A majority with CSF pleocytosis had lymphocytic predominance in the CSF and only one patient had neutrophil predominance. Median CSF protein concentration was 72.6 mg/dL (range 24.1 – 157.4) in the neuroleptospirosis group and the CSF: blood glucose ratio varied from 0.15 to 0.90 (median 0.57). All patients (except one with a high CSF cellular reaction of 730 cells/μL) had neutrophils <30/μL and lymphocytes <100/μL in the CSF (Table 3). There were two distinct subgroups in patients identified as neuroleptospirosis. One subgroup (n = 4) had positive serum and/or CSF *Leptospira* DNA suggestive of early infection (Table 3). Majority in this subgroup had acellular CSF (3/4, 75%) with normal protein levels (3/4, 75%). The second subgroup had positive MAT (n = 7), and a majority in this group had a cellular reaction in CSF (n = 6, 85.7%) and elevated CSF protein (n = 5, 71.4%). Median CSF: blood glucose ratio was higher in patients who had positive blood or CSF PCR compared to the group who had positive MAT (0.67 vs 0.45, p < 0.05).

## Discussion

Primary neuroleptospirosis is rare and leptospirosis is not considered in the routine laboratory workup of primary CNS infections. Our multicentre study conducted in three areas of high leptospirosis burden in Sri Lanka identified 11 (11%) neuroleptospirosis cases among patients presenting with features of a primary CNS infection, cases that would not otherwise have been suspected, emphasizing the importance of considering leptospirosis in the differential diagnosis. Clinical presentation of neuroleptospirosis is similar to other CNS infections except for the significant occurrence of focal neurological deficits such as speech involvement and limb weakness, thus necessitating laboratory investigations to arrive at the diagnosis. Although leptospirosis classically gives rise to thrombocytopaenia, cholestatic hepatitis and AKI [5], baseline laboratory investigations did not show a significant difference in the two groups at initial presentation. Development of AKI, hepatitis and thrombocytopaenia during the course of illness may be a clue to the diagnosis of neuroleptospirosis, as was seen in four of our patients. Treatment received by the neuroleptospirosis group was similar to the group with other CNS infections. Five out of the 11 patients received steroids, as part of initial empiric therapy of suspected bacterial infection to reduce neurological sequalae. Outcome in patients with neuroleptospirosis who received steroids was similar to those who did not in our study. Further studies are required to identify the optimum treatment, including the role of steroids, in neuroleptospirosis. Studies delving into the pathogenesis of neuroleptospirosis would shed light on the most appropriate management.

Importantly, patients with primary neuroleptospirosis in our cohort, had a favourable outcome with no permanent neurological disability, and only one patient required escalation to intensive care. Our data is comparable to findings from a prospective study in Laos which demonstrated similar outcomes [25].

In contrast, a study from India which primarily recruited patients with hepato-renal involvement reported mortality of 26% (n = 8/31); all these patients had positive MAT, presented in the immune phase and had hepato-renal involvement [10]. Outcome could be significantly affected by other organ involvement such as renal and liver. In addition, differences in strains and host factors could account for these varied manifestations and outcomes, highlighting the importance of local epidemiological data on geographical variations in *Leptospira* serovars.

A diverse range of neurological manifestations are reported in leptospirosis which vary from aseptic meningitis [12,20,22,24], acute disseminated encephalomyelitis [34–36], cerebritis [37], autoimmune encephalitis [38,39], subarachnoid haemorrhage [40] to cerebral arteritis [13,41]. Most of these manifestations occur in the immune phase of illness following the initial leptospiraemic phase. Our findings reveal two distinct phases of neurological involvement in leptospirosis. One group (n = 4) had positive serum and/or CSF *Leptospira* DNA, suggestive of early infection. This group had minimal CSF inflammation as evidenced by acellular CSF (n = 3, 75%) with normal protein levels (n = 3, 75%). The second group had positive MAT (n = 7) suggestive of presentation in the immune phase and showed features of aseptic meningitis. Although the pathophysiology of primary neuroleptospirosis is poorly understood, our findings suggest that it may result from direct bacterial invasion as well as host immune response [7]. Leptospira are known to reach CSF and the CNS within 48 hours of introduction [7]. Although most manifestations are attributed to endothelial damage and vasculitis [7], initial leptospiraemic phase elicits minimal CSF inflammation. The sensitivity of PCR used (Lipl32 gene) in the reference laboratory is approximately 67% with a specificity of 90% and it detects pathogenic *Leptospira* species [42]. The two patients with positive PCR and labelled as “probable meningitis” had a clinical syndrome suggestive of meningitis, although there was no CSF pleocytosis. This could be due to early meningitis as described previously in bacterial meningitis [43]. We acknowledge that, outside of CSF PCR positivity, attribution to leptospiral CNS infection is inferential; therefore, we interpret our MAT-based confirmations cautiously and present them as supportive of neuroleptospirosis in the clinical context.

Patient 7 had a prolonged duration of symptoms (15 days) at the time of presentation with positive serum PCR for *Leptospira* but a negative MAT result. Although serological positivity is generally expected during the immune phase of leptospirosis, delayed or absent seroconversion has been reported in some patients. Several explanations may account for this finding. First, the timing of antibody production may vary between individuals, and MAT seroconversion may occur later in the disease course in some cases. Second, early antibiotic therapy prior to presentation may suppress the humoral immune response and delay detectable antibody production. Third, MAT panels detect antibodies against a limited number of serovars, and infections caused by antigenically divergent strains may result in reduced MAT reactivity. Finally, PCR may detect circulating leptospiral DNA even when viable organisms are no longer present, which could account for PCR positivity despite negative serology.

There is little data on CNS infection in leptospirosis, especially without other features of leptospirosis (primary neuroleptospirosis). The available literature is limited to mainly case reports [12,21,23,24] and a few case series [22]. The published observational studies were conducted in South Asia, Southeast Asia, Sudan and Brazil with no published data from other regions of the world [7,10,26–29]. Panicker et al. described primary neuroleptospirosis in 40 patients in a study over 4 years in South India; 16 of them had evidence of CNS infection (13 aseptic meningitis, 3 meningoencephalitis) [9]. A study in Laos was aimed at identifying multiple pathogens in CSF samples of patients undergoing lumbar puncture which identified leptospirosis as an aetiological agent in 2.9% of participants [25]. In a series of 100 cases of CNS infection from Philippines, 5 patients with leptospirosis were identified [26]. Interestingly, a four out of 19 cases of leptospirosis in Netherlands had meningitis; all of them had a history of travelling in Southeast Asia [22]. These data are comparable to our finding of 11% neuroleptospirosis among patients with a primary CNS infection. However, two studies from Brazil by Romero et el. reported positive CSF *Leptospira* DNA in 40–60% of patients with aseptic meningitis [27,28]. These patients were selected based on negative traditional microbiological tests and predominant lymphocytes in CSF.

Our study is among the first from South Asia to systematically examine neuroleptospirosis as a cause of CNS infection using both molecular and serological diagnostics. We believe our findings add to the limited literature on this important clinical entity, and highlight the need for increased awareness. They have important implications for development of diagnostic algorithms and clinical management protocols in endemic settings. Our study was conducted in three regions of Sri Lanka with high leptospirosis disease burden, limiting the generalizability of the findings. These areas are documented to have an increase disease burden due to occupational exposures such as farming, gem mining and exposure to frequent water sources during rainy seasons [44]. It is not known whether the prevalence of primary neuroleptospirosis would be similar in other regions of the country, and in other tropical countries. Another limitation was the small number of confirmed cases of neuroleptospirosis, but this was comparable to the few published case series [10,25]. The sample size caused limitations in interpreting the effect of treatment such as steroids. However, the sample size was adequate to give important inferences, highlighting the need to expand the laboratory workup in CNS infections to include leptospirosis. We did not have neuroimaging findings in all patients. Although imaging is not essential in arriving at a diagnosis of a CNS infection, contrast-enhanced CT or MRI scans would have added important information on CNS inflammation, especially any focal involvement; this would have been useful in correlation of the focal symptoms noted in the leptospirosis group. MRI scans are not available in most healthcare settings in Sri Lanka and its availability is restricted to a few referral centres. Lower-middle income countries endemic for leptospirosis face the same scenario when investigating neuroleptospirosis and hinder true definition of this syndrome. A future study focusing on the imaging abnormalities of neuroleptospirosis could provide important information to fill this gap. Routine investigations of primary CNS infections in this cohort included pyogenic culture, direct smears in the CSF and Herpes simplex virus PCR in CSF. Our study was not designed to identify potential aetiological agents other than *Leptospira* and the rare occurrence of dual infections could have been missed in this cohort. It is plausible that other pyogenic infections (e.g.,: patient 3 having co-existing otitis media) or viral or atypical infections occurring as co-infections could have been missed due to the study not designed to explore all infections. In addition, the study was not designed to identify and describe the aetiological agents of the non-neuroleptospirosis group which would have added further information during comparisons. Sri Lanka is a South Asian country, and it would be interesting to see the geographical distribution of primary neuroleptospirosis worldwide. A multicentre surveillance in leptospirosis endemic countries would be useful to identify the true burden of primary neuroleptospirosis.

## Conclusions

Primary neuroleptospirosis is an important but easily overlooked cause of primary CNS infections. Initial clinical presentation or basic laboratory investigations are not helpful in distinguishing neuroleptospirosis from other CNS infections, necessitating a high degree of clinical suspicion and specific microbiological investigations for an accurate diagnosis. Primary neuroleptospirosis presents in the initial leptospiraemic phase as well as in the immune phase of the infection and carries a good prognosis. Neuroleptospirosis should be considered in the differential diagnosis of primary CNS infections in endemic settings.
